# Perceptions, intentions, and actual use of a consumer nicotine gum

**DOI:** 10.1186/s12954-023-00864-0

**Published:** 2023-09-13

**Authors:** Cheryl K. Olson, Neil Sherwood, Maia Berkane, Karin Gilligan, Willie J. McKinney

**Affiliations:** 1 Cheryl K. Olson, Sc.D., LLC, 633 Elm Street, San Carlos, CA 94070 USA; 2 Neil Sherwood Consulting, Avenue Alfred Cortot 21, Nyon, 1260 Switzerland; 3 Kevin D. Oden & Associates, LLC, 505 Montgomery Street, San Francisco, CA 94111 USA; 4 McKinney Regulatory Science Advisors, LLC, 4940 Old Main Street, Unit 603, Henrico, VA 23231 USA

**Keywords:** Tobacco, Harm reduction, Nicotine gum, Perception and intention studies

## Abstract

**Background:**

Little is known about perceptions, use intentions, and behaviors of adults regarding nicotine gum that is marketed and regulated as a consumer product rather than as a medicinal nicotine replacement therapy (NRT).

**Methods:**

Survey data were collected from a Qualtrics online panel (*N* = 1000) of adults who had never used a consumer nicotine gum, recruited based on smoking behavior, and from current and former purchasers of one commercially available nicotine gum product (LUCY Chew and Park), recruited via emails to a customer database (*N* = 500). In addition to descriptive cross-sectional analyses, logistic regression was used to estimate the probability of intent to try and of product appeal among these different groups.

**Results:**

Among online panel respondents, individuals who smoked with and without plans to quit showed high intention to try the product (odds ratios 15.6 [95% CI 9.3, 27.6] and 9.8 [95% CI 5.8, 17.3] respectively, compared to people who formerly smoked) and persons who had never smoked showed low intentions to try. These results stood regardless of flavor. Among current and former purchasers of the study product, 43.4% of persons who had smoked cigarettes regularly indicated they were motivated to try the product “to help me quit smoking.” Only 0.6% of young adult consumers of the nicotine gum (aged 21–30) had not tried tobacco products previously.

**Conclusions:**

Consumer nicotine gum does not appear to attract those who have never used a tobacco product and the results for young adults suggest minimal appeal to youth. The study product was used primarily by individuals who currently smoke and/or use e-cigarettes but who wished to quit or reduce consumption. These results suggest that a consumer nicotine gum may reduce harm by substituting for higher-risk products such as combustible cigarettes.

## Background

Worldwide, cigarette smoking remains one of the primary preventable risk factors for disease and premature death. A systematic analysis of data from the Global Burden of Disease Study 2019 found that more people are smoking today than ever before. This is due to a combination of increased smoking rates in some nations, population growth outstripping decreased smoking prevalence in others, and stalled progress in nations that previously saw drops in smoking rates [1]. The authors noted that up to two-thirds of people who smoke cigarettes long-term will die of a smoking-attributable disease.

Evidence suggests that currently available approaches to smoking cessation are not adequate to this task. A population impact model of the effects of nicotine replacement therapy (NRT) products, varenicline, and bupropion [2] found that using these recommended methods, just 2.3% of persons who smoke would quit, equaling a reduction in United States (U.S.) population-level smoking prevalence of 0.3%.

Abstinence from all tobacco products is the most beneficial to health, but may not be a realistic goal for everyone. Regulators and researchers view tobacco products as falling along a continuum of risk. For adult tobacco consumers, combustible cigarettes are a far more hazardous nicotine delivery system than various forms of non-combustible nicotine [3]. Tobacco harm reduction, initially viewed skeptically by many in the tobacco control community, is increasingly accepted as a means to rapidly reduce disease and death for people who smoke [4].

This is compatible with the human-rights approach to harm reduction used with other drugs [5]. Applying these principles to consumers of nicotine encourages a focus on positive change rather than judgment or coercion. Given the limited effectiveness of reduced-harm nicotine products labeled and regulated as medicines, consumer-oriented reduced-risk nicotine alternatives may have a meaningful role in promoting positive change. As a recent international review noted [6], countries with relatively higher adoption rates of alternative nicotine products typically not designated as cessation aids (e.g., snus, e-cigarettes and heated tobacco) have lower smoking rates.

The last decade has seen an increasing number of tobacco and nicotine-containing products introduced to the U.S. market that expose adult tobacco consumers to significantly fewer of the known cigarette smoke toxins. These new products include e-cigarettes, heated tobacco products, oral tobacco products, and modern oral nicotine products [7, 8]. These tobacco and nicotine-containing products are not marketed as medicinal NRTs, which are aids to smoking cessation, but are marketed as consumer packaged goods that are intended to provide adults who smoke combustible cigarettes with less harmful satisfying alternatives.

Notably, the U.S. Food and Drug Administration (U.S. FDA) has stated on several occasions that switching to new tobacco and nicotine-containing products that reduce the exposure of adults who smoke to Harmful and Potentially Harmful Constituents (HPHCs) such as tobacco-specific nitrosamines (*N*′-nitrosonornicotine [NNN] and Nicotine-Derived Nitrosamine Ketone [NNK]) may reduce the harm associated with smoking [9]. For example, over the past 25 years smoking rates and the incidence of smoking-related diseases in Sweden have declined greatly relative to other nations, with some credit given to the widespread uptake of the low-nitrosamine containing oral tobacco product snus, especially among men who smoked cigarettes [10]. Studies have found that snus is more appealing to people who smoke and can be more effective at achieving the goal of smoking cessation than NRTs [11, 12].

These observations have increased interest in the potential of other smokeless tobacco products to reduce harm by helping persons who smoke highly toxic combustible cigarettes reduce the number smoked, quit smoking, or quit all tobacco consumption. However, harm reduction must be accomplished without causing significant uptake by those naïve to tobacco products, particularly youth [11, 13–15]. Supporting data are considered essential in demonstrating that a tobacco product is “Appropriate for the Protection of Public Health” (APPH), the relevant product standard for tobacco products employed by the U.S. FDA. Currently, additional research is needed on other novel, potentially reduced-risk smokeless products that may appeal to and switch consumers of combustible tobacco products [4], especially women and persons who are not actively trying to quit.

Nicotine gums approved by the U.S. FDA as NRTs, with a HPHC profile far superior to combustible cigarettes [16], have been available in the U.S. for 25 years [17] and have an excellent safety record [18]. Only one published study that included information on perceptions and use behaviors regarding nicotine gum not marketed as NRT was found [19]. The cross-sectional U.S. online panel survey on oral nicotine products included 6131 subjects aged 13 to 40. Among subjects under 21, 20.9% said they had tried any oral nicotine product, and 12.2% had tried any nicotine gum; 45.6% of subjects 21–40 had tried an oral product and 29.6% tried nicotine gum. Cigarette consumption history was not reported.

Data on perceptions of one brand of non-NRT gum (LUCY) were collected from a random subset of 2730 subjects. Consumption data were provided for that subset only. The data table shows that 4.2% of those under 21 and 12.4% of those aged 21–40 said that they had tried the product. The stimulus for perception questions was a screenshot of the home page of the product website. Based on this advertising, subjects were asked how much they liked the product and how likely they were to buy. Among subjects under 21, 57.4% liked the gum “not at all”; 70% were “not at all” or “very unlikely” to buy it. For ages 21–40, 67.3% were not at all or very unlikely to buy. Disinclination to buy was slightly greater for those who had ever tried the product, in both age groups.

In the current publication, we share the results of two studies of one brand of commercially available consumer nicotine gum: a perception and intention study of an online panel of adults naïve to the study product (segmented by smoking behavior), and a perception, intention, and “actual use” study of adult current and former study product purchasers. Similar to a recent study of novel smokeless products [20], the surveys utilized for this study assessed appeal and consumption intentions, as well as reasons for initiation and patterns of consumption among adults with various tobacco use experiences. In addition, we assessed how results differed for subgroups such as people intending to quit and not intending to quit smoking, for women (who show low interest in traditional smokeless tobacco products, such as snus), and for persons who had previous experience with NRT gum. We further assessed whether the product might appeal to people who had never smoked, including youth, or to people who formerly smoked. This included assessment of how results might differ by flavor, given research and concerns about how flavor may affect appeal and abuse liability for youth and for persons who smoke [21].

Finally, we collected open-ended comments from all study participants to better understand factors that encourage or impede trial or continued consumption (including flavor, texture, and branding) and to obtain examples of possible behavior change trajectories. These included comments comparing experiences with this consumer nicotine gum to a NRT gum, to understand what distinctive niche a consumer nicotine gum might occupy on the tobacco product continuum of risk [3].

## Methods

LUCY Chew and Park, the product under study, is manufactured and marketed in the U.S. by Lucy Goods Inc. Each piece contains 4 mg of nicotine bound to an ion-exchange resin (polacrilex), and is intended for and marketed to adult tobacco consumers for nonmedicinal or nontherapeutic use.

We recruited and conducted online surveys of two categories of subjects: adults who had tried/used the product (*N* = 500) and those who had not (*N* = 1000). These sample sizes allowed us to further differentiate among key subsamples.

### Online panel sample quotas and recruitment

Survey respondents naïve to the product were recruited in March 2020 from a Qualtrics nationwide sample, drawn from double-opt-in market research panels. Survey respondents received incentives (e.g., cash, gift cards, airline miles) that varied based on survey length and panel member profile (i.e., acquisition difficulty). Recruiting criteria for the desired 1000 product-naïve subjects in the sample included: adults over age 21 (roughly 30% ages 21–34, 40–50% ages 35–54, and 20–30% ages 55+); never tried the product; equal numbers of subjects (*N* = 250) in each of four smoking behavior categories (see below); and roughly equal numbers of males and females.

Smoking behavior categories and screening questions were derived from questions on tobacco product use from the National Institutes of Health’s Health Information National Trends Survey (HINTS) [22]. Because people participating in online panels are paid per survey (and may seek to provide the “right” answer to gain entry), screening questions were neutrally phrased to discourage guessing or misrepresentation, and multiple responses required for assignment to a smoking behavior category.

The four smoking behavior categories were: (1) never smoked (persons who smoked fewer than 100 lifetime cigarettes and do not smoke at all currently); (2) formerly smoked (persons who had completely quit combustible cigarettes more than 1 month earlier); (3) smokes—intends to quit (smoked daily and was either “trying to quit now” or “seriously considering” quitting within the next 6 months); and (4) smokes—no intent to quit (smokes daily and was neither “trying to quit now” nor “seriously considering” quitting within the next 6 months).

Recruiting for the subgroups of persons who currently and formerly smoked cigarettes was capped by age, so that no more than half would be over age 55.

No identifying information was collected (such as name, location, or contact information) and age was requested as a range only.

### Consumer nicotine gum survey quotas and recruitment

The recruiting goal was 500 valid and completed surveys from U.S. adults over 21 who had experience with the product, including 300 current consumer nicotine gum purchasers (last product experience within the past month) and 200 former purchasers (last product experience 1 month to more than a year ago). Up to fifty recruits were considered if they only tried the product once or twice (within the past 6 months) in order to capture the views of people who chose not to continue consuming the product. Based on the manufacturer’s estimates that a quarter of purchasers to date had been female, the quota for males was set at 300–350 and females at 150–200.

Subjects were recruited from the manufacturer’s database of customers who had purchased a product on their e-commerce retail store (http://lucy.co), as well as through email addresses submitted by potential customers who browsed the website and received marketing emails. All website visitors self-certify that they are over the age of 21 and are subject to full age verification upon making a purchase. The customer list was geographically diverse, comprising all 50 states and only modestly weighted toward the population-dense regions of the Northeast and West Coast.

Purchaser survey subject privacy was protected in multiple ways. Survey data collected did not include identifying information (no location, age as part of a range, etc.) and were not linked in any way to individuals in the email database. Subjects who completed the survey were sent to a separate company to claim the gift card incentive via email. Recruitment emails noted that feedback would be kept anonymous and confidential, and that participation would not result in contact by the manufacturer or anyone else.

## Measures

Research on novel tobacco products such as e-cigarettes has shown a link between favorable perceptions of products and product consumption, as well as unfavorable perceptions to non-consumption or cessation. However, there is limited consensus on the best ways to measure perceptions of product risks and benefits [23, 24]. As Gibson et al. suggest, comparing novel products to widely known and better-studied products such as cigarettes and NRTs may also clarify motivations for choosing one product over another [23].

Accordingly, to address the goals and research questions above we looked for examples and guidance on best practices in recent published research and reviews as well as in previous industry applications to the U.S. FDA and the FDA’s own guidance [25]. For measuring risk perception and relative risk, we followed recommendations to construct response options using verbal qualitative comparisons (e.g., from ‘no risk’ to ‘very high risk’) rather than numerical scales from 0 to 100% [26], and used a combination of rating and ranking tasks (including a spectrum of tobacco products) to reduce social desirability bias. When measuring perceptions of product addiction risk, we addressed mood states (e.g., “having to smoke cigarettes to feel better”) as well as physical need [27].

We also looked at short-term and longer-term health consequences (e.g., frequent minor illnesses, earlier death), since the salience of these consequences are thought to vary by age, and included “I don’t know” options to questions where uncertainty might otherwise bias responses (e.g., when facts are not established, or when subjects are asked to estimate the views of others) [23]. Product appeal was assessed by flavor (wintergreen, cinnamon, pomegranate). When assessing product appeal, we addressed whether each flavor’s packaging would “appeal to people your age” as well as “appeal to someone like you,” given research on the role of perceived “smoking identities” in youth use and the history of tobacco product advertising targeted by demographic and psychosocial factors.

Following recommendations to address intention to use a novel tobacco product in multiple ways, we assessed: intent to try it, intent to employ it as an aid in cessation of all tobacco consumption, and intent to use it concurrently with other tobacco products [27]. A question on intent to try (and the concept of “positive intention to try/use”) was based on Philip Morris International’s (PMI) Intent to Use Questionnaire [28], which was applied in assessing intent to use PMI’s IQOS product, drawing upon U.S. FDA’s 2012 draft guidance for Modified Risk Tobacco Products. For ease of reading, greater detail is provided on wording of some questions where results are presented (below).

To increase validity and minimize confusion, questions were worded to match the everyday language used by the general public when talking about tobacco products. For that reason, the surveys described the product as a “nicotine gum” and used Nicorette® as a generally understood stand-in for NRT gum, as has been done in other surveys (e.g., the National Health Interview Survey) [29].

Questions on warning comprehension, product consumption, motivations for trying, and product satisfaction/dissatisfaction drew upon customer responses to previous marketing research and on the manufacturer’s interactions with customers over time, as well as research on NRT gums [17, 30]. Questions on tobacco consumption and quitting also drew upon the National Cancer Institute’s HINTS survey.

Some open-ended questions were included to help capture narrative details about consumers’ actual use of the product. These were analyzed based on frequency and tone of spontaneous mentions of various topics or issues, and representative examples of consumer perceptions of the product in their own words are provided. Many questions allowed more than one response and were marked “select all that apply.” Some questions were presented only to subsets of subjects (e.g., current product purchasers; people who smoked; people who had used Nicorette®).

To better understand how the sample compares to the U.S. adult population, data were also collected from both samples on race/ethnicity, income level, and education level.

### Cognitive interviews

Both surveys were formally pre-tested to assess comprehension of item intent and phrasing and ease of responding, with individuals drawn from the same subject pools as survey respondents. To preserve social distancing during COVID-19 lockdowns, subjects used their home computers to complete the surveys, reporting any areas of confusion or difficulty via audio link.

Changes were made along the way and tested with subsequent participants. For example, in the online panel survey’s product use description—although similar to the Nicorette® package insert—the term “park” was confusing to several subjects and associated with chewing tobacco. Prefacing the use description with “like other nicotine gums” made it clear that the product was not a form of chewing tobacco.

### Quality checks

All survey questions required a response to move forward. If open-ended questions received a nonsensical or random-letters response (suggesting a subject rushing to claim an incentive), all responses from that subject were deleted as invalid.

### Statistical analysis

Because this was a descriptive study, most results are presented in the form of percentages and frequencies among subgroups of interest. We calculated odds ratios to determine the probability of intent to try any product flavors (and of personal product appeal) among subgroups with different smoking behaviors. Intent to try was recorded as 1 if a “positive intention to try” (“very likely” or “definitely will” try) any product flavor, and 0 otherwise. Because this output is binary, ordinary least squares regression was not appropriate; instead, logistic regression was used. People who formerly smoked were used as the reference group, and other smoking behavior subgroups compared to that in terms of odds of intention to try.

## Results

### Participant characteristics

#### Online panel sample (naïve to study product)

This recruited sample of 1000 was evenly divided by gender, and included equal numbers of persons who never smoked, who formerly smoked, who smoked but intended to quit, and who smoked but did not plan to quit. As a group, those who never smoked were younger (49.2% under age 35, and 32.4% age 45 or older) and those who formerly smoked were older (24.0% under 35, and 59.6% age 45 or older). Participants who smoked with intent to quit and no intent to quit had a similar age distribution at the younger end (28.4% and 30.4% under 35 respectively), but persons not planning to quit were more likely to be older (48.0% age 45 or older) than persons planning to quit smoking (37.2% age 45 or older). Participants who had never smoked skewed male (77.2%) and those who formerly smoked skewed female (68.4%). Participants who smoked were more evenly balanced, with 54.4% identifying as female and 45.6% as male in both categories.

Nearly three-quarters (73.2%) of participants had attended college. Those who currently smoked had lower levels of educational attainment than other groups. Among those who had never smoked, 49.2% had obtained a college degree or higher, compared to 35.2% of those who formerly smoked, 25.2% of persons intending to quit smoking, and 20.4% of persons not intending to quit. Over half of respondents (52.4%) reported household incomes under $50,000; a greater proportion of persons not intending to quit (63.6%) fell into this category compared to persons who had never smoked (39.2%). Concerning race/ethnicity (where participants could select multiple categories), 78.2% identified as white, 11.6% as black/African American, 4.4% Hispanic/Latino (any race), 4.3% Asian/Indian/Pacific Islander, 0.5% Native American, and 1% as “other.” There were relatively fewer white respondents (64% of total) among persons who had never smoked, and relatively more among those who formerly smoked (90% of total).

#### Consumer nicotine gum purchaser sample

The sample of 500 people exposed to the study product included 294 current purchasers, 186 former purchasers, and 20 who had tried the product once or twice. The sample was 69.4% male (*N* = 347) and 27.6% female (*N* = 138). (Ten subjects identified as non-binary, and 5 chose “prefer not to say.”) Respondents could choose more than one racial/ethnic category. Most respondents (88.4%) identified as white; 5.6% selected Asian/Indian/Pacific Islander, 5% Hispanic/Latino, 2.6% black/African American, 1.8% Native American, and 1.2% Middle Eastern/North African, with 3% selecting “other” or “prefer not to say.” Asked about household income, 24.2% reported an income of under $50,000, and 25.2% reported $50,000–$74,000; the remainder reported $75,000 or a higher category. The large majority had attended college (92.4%), with 56.8% earning a 4-year degree or beyond.

Two-thirds (61.6%) of the purchaser sample had vaped regularly, and half (49.8%) had smoked cigarettes regularly before starting the study product. Only 0.6% of young adult respondents (aged 21–30) had not tried or regularly consumed tobacco products before trying the consumer nicotine gum.

### Tobacco product perceptions and intentions among online panel

#### Warning comprehension

**Fig. 1:**
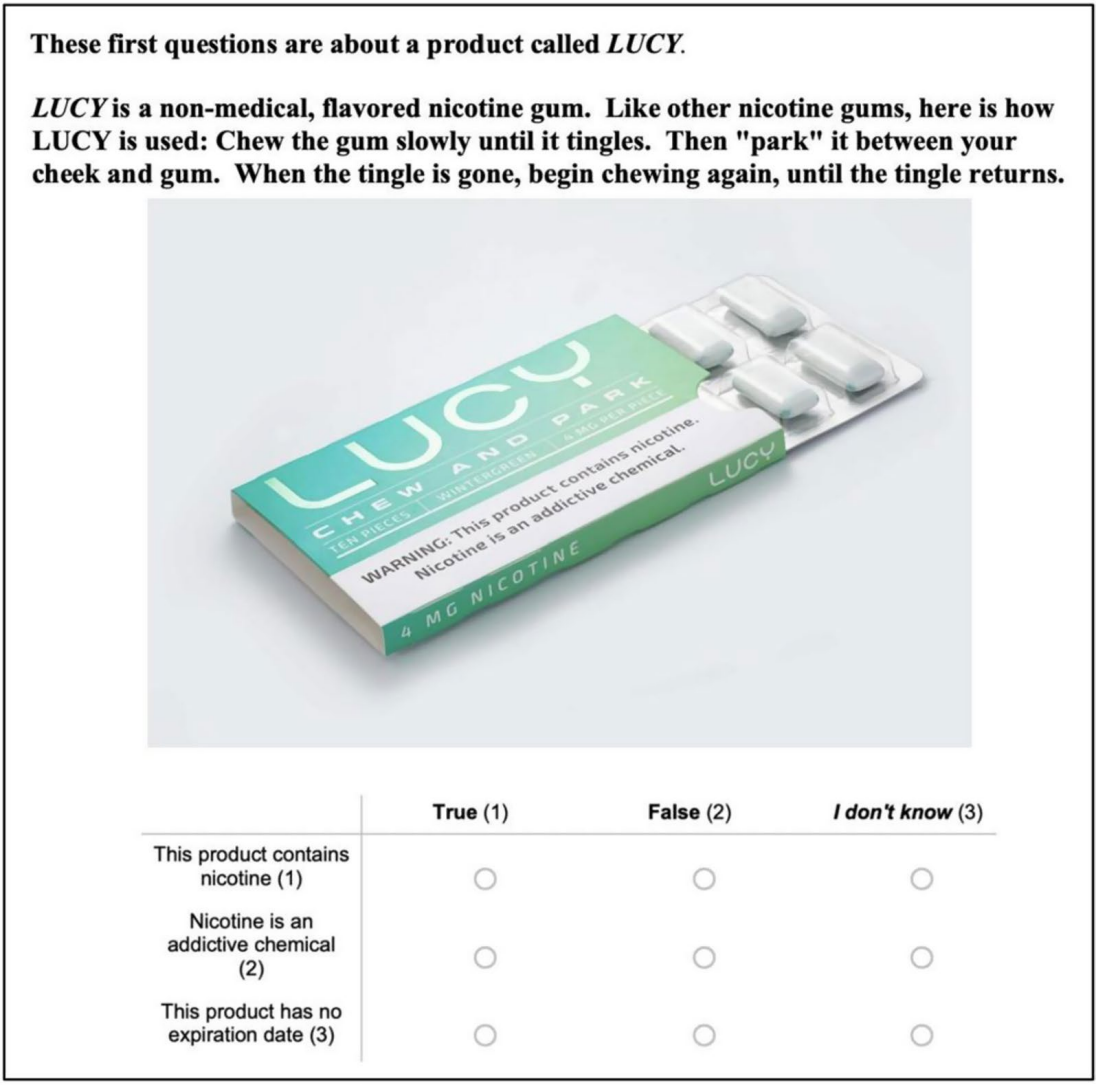
Product description and package image

To orient respondents to the novel product, a brief description was provided, along with a package image (current as of March 2020) showing the warning label (Fig. 1).

“This product contains nicotine” was marked “True” by 93.2% of all 1000 respondents, “False” by 2.6%, and “Don’t Know” by 4.2%. “Nicotine is an addictive chemical” was marked “True” by 94.2%, “False” by 2.6%, and “Don’t Know” by 3.2% of all subjects. (A third question on expiration date was intended as an attention check, to ensure that there was not an issue with online survey respondents rushing or guessing; a date was not given.)

#### Perceived product appeal to peers, by smoking behavior and flavor

The online panel perception and intention study also included questions to assess perceptions of and behavior intentions for each product flavor. Subjects were presented in turn with a large image of the packaging for each of the three product flavors: wintergreen, pomegranate, and cinnamon. Flavors were shown to respondents in a rotating order to allow the option of assessing presentation-order effects.

A series of three questions was asked about each flavor, based on that image. Subjects were asked about the appeal of that product to people their age, followed by the product’s appeal to themselves, and finally about their intention to try that product.

To the question, “In your opinion, would [study product brand name] nicotine gum [flavor] appeal to people your age?” response options were “Definitely not,” “Very unlikely,” “Somewhat unlikely,” “Somewhat likely,” “Very likely,” “Definitely,” and “I don’t know.”

To facilitate easy comparison of smoking behavior subgroup responses, we combined the top two categories to create a simple measure of perceived peer appeal. Positive age group appeal was defined as a response that a flavor would “definitely” or “very likely” appeal to people of the respondent’s age.

**Fig. 2:**
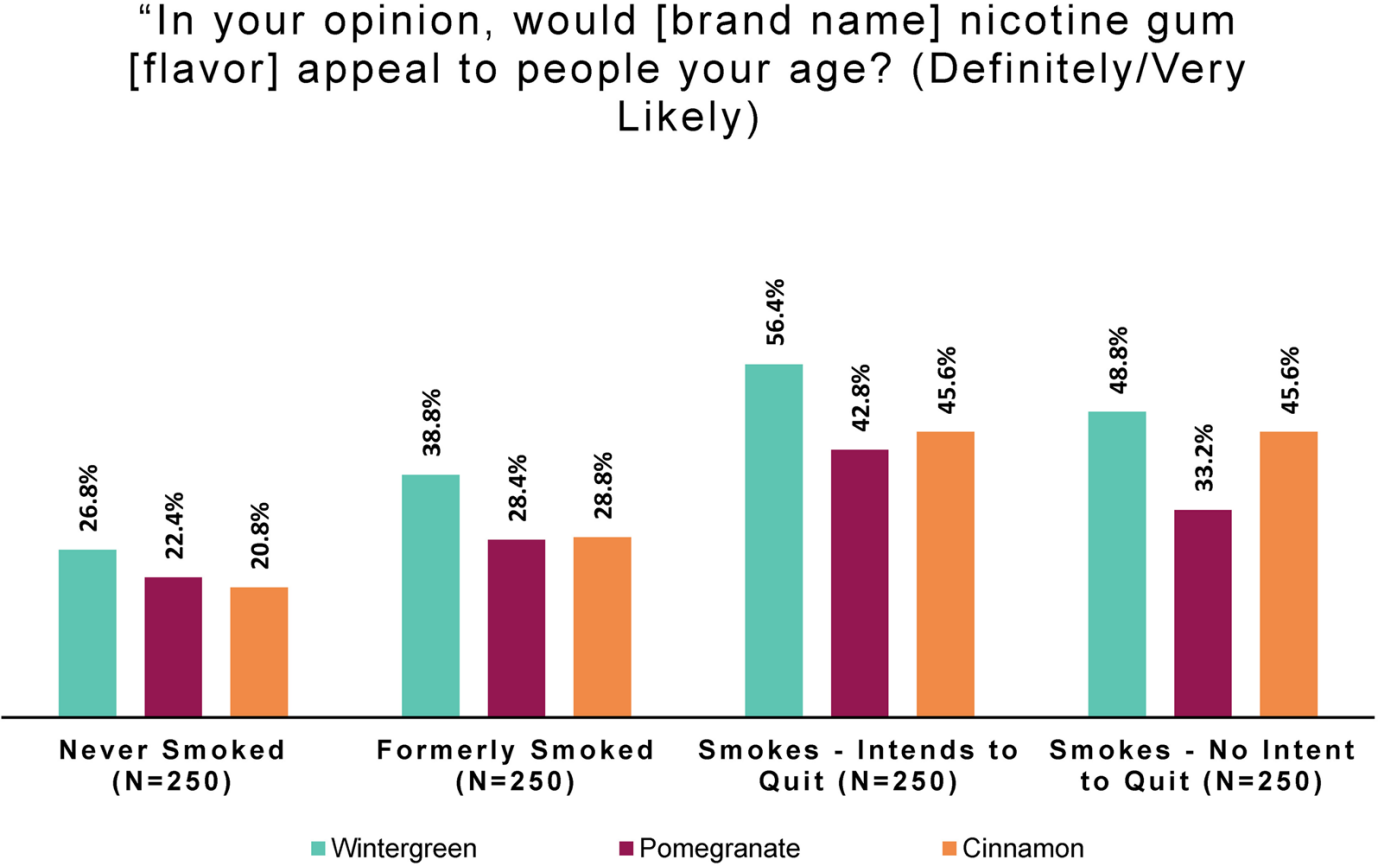
Positive age group appeal: by smoking behavior and flavor (online panel)

As Fig. 2 shows, comparatively few persons who had never smoked perceived the study product as appealing to peers. Only a minority of persons who formerly or never smoked viewed any flavor as positively appealing to people of their age group.

#### Perceived personal product appeal, by smoking behavior and flavor

Asking whether this product was “designed to appeal to someone like you” revealed a large difference between persons who did and did not smoke, beyond that seen for peer appeal. (Response options were: “Definitely not,” “Probably not,” “Possibly not,” “Possibly,” “Probably,” and “Definitely”.)

For ease of comparison of subgroup responses, we again combined the top two categories to create a simple measure of perceived appeal to oneself. Positive personal appeal was defined as selecting “probably” or “definitely” regarding “appeal to someone like [the respondent].”

**Fig. 3:**
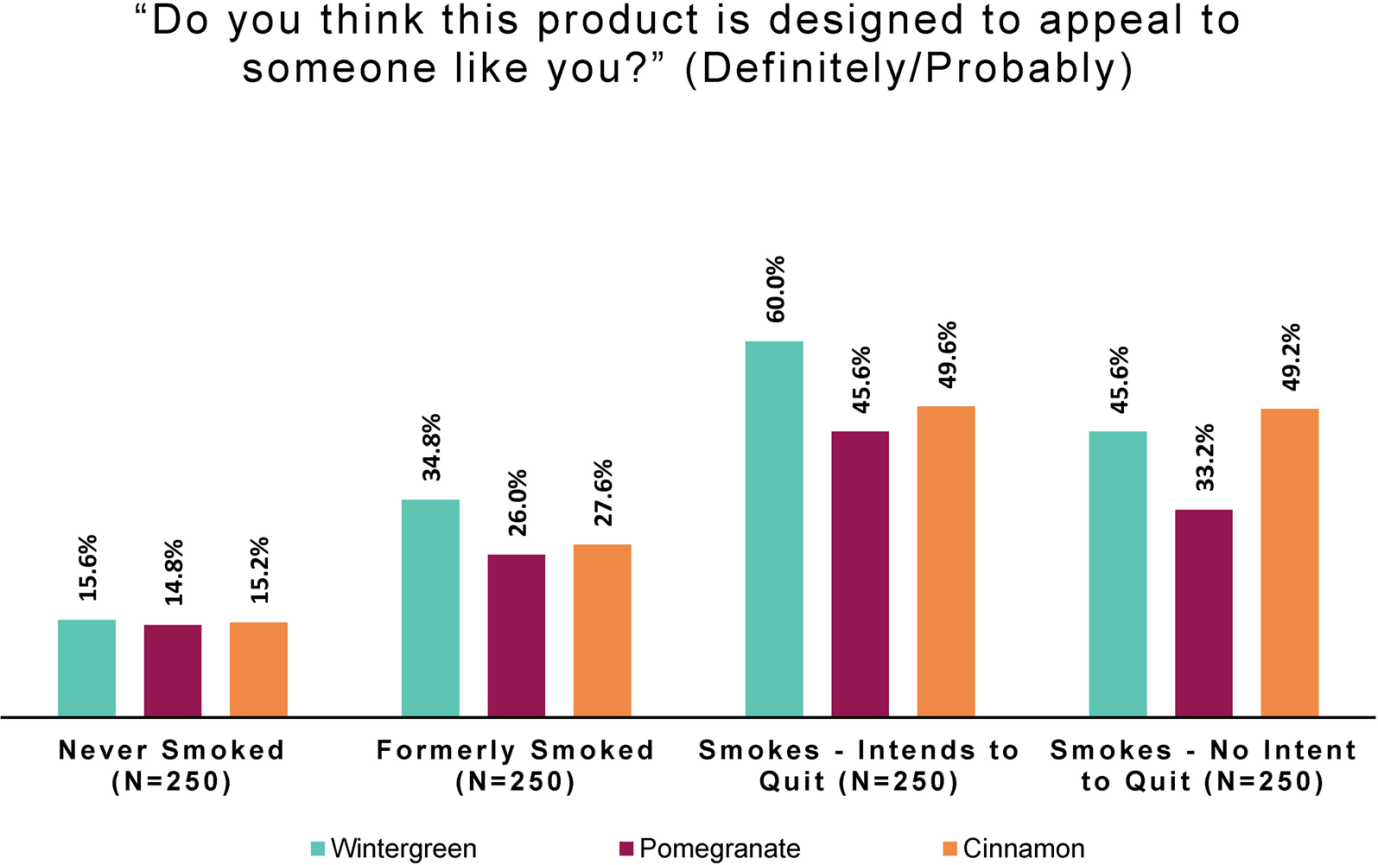
Positive personal appeal: by smoking behavior and flavor (online panel)

As seen in Fig. 3, the study product disproportionately appeals to persons who currently smoked; roughly half find at least one flavor appealing. By contrast, few of those who had never smoked (roughly one in seven) or no longer smoked found any flavor appealing. In fact, among persons who never smoked, “definitely not” was the most common response: 38.8% for wintergreen, 42.8% for pomegranate, and 40.8% for cinnamon.

Logistic regression was performed to express the odds of finding any product flavor appealing for each smoking behavior group, using persons who formerly smoked as the reference group. As shown in Table 1, those who responded “probably” or “definitely” to at least one of the flavors were scored as “yes” on positive personal appeal. Table 2 shows the results of the logistic regression of this combined personal appeal variable on smoking behavior status. The odds of combined study product flavors appealing to persons who formerly smoked are 0.75 (1.3 times the odds of no appeal); the odds of appeal to persons who never smoked are half those of persons who formerly smoked. The odds of appealing to persons not intending to quit smoking and persons intending to quit are 2.2 times and 3.7 times, respectively, those of persons who formerly smoked.

**Table 1:** Positive personal appeal of any study product flavor, by smoking behavior

Smoking behavior	No	Yes	Total
Formerly smoked	143 (57%)	107 (43%)	250 (100%)
Never smoked	186 (74%)	64 (26%)	250 (100%)
Smokes-no intent to quit	94 (38%)	156 (62%)	250 (100%)
Smokes-intends to quit	67 (27%)	183 (73%)	250 (100%)
Total	490 (49%)	510 (51%)	1000 (100%)

**Table 2:** Logistic regression analysis of positive personal appeal of any study product flavor

Smoking behavior	OR	95% CI	*p* value
Formerly smoked	–	–	
Never smoked	0.46	0.31, 0.67	< 0.001
Smokes-no intent to quit	2.22	1.55, 3.18	< 0.001
Smokes-intends to quit	3.65	2.52, 5.34	< 0.001

*OR* odds ratio, *CI* confidence interval

#### Product intention to try by smoking behavior and flavor

To understand intentions to try the product, subjects were asked, “How likely or unlikely are you to try [study product brand name] nicotine gum [flavor]?” (Response options were: “Definitely will not try,” “Very unlikely to try,” “Somewhat unlikely to try,” “Somewhat likely to try,” “Very likely to try,” and “Definitely will try”.)

Looking at smoking-behavior subgroup responses regarding behavior intentions, we observed an even larger difference among persons who did and did not smoke than we saw in perceptions of appeal. Few persons who never smoked or formerly smoked intended to try any study product flavor. The large majority of persons in these groups “definitely will not try” or are “very unlikely to try” wintergreen (78.0% of those who never smoked, 78.4% who formerly did), pomegranate (80.8% who never smoked, 82.8% who formerly did), or cinnamon (79.6% who never smoked, 81.6% who formerly did). In other words, persons who never or formerly smoked displayed what might be called a high negative intention to try any product flavor.

**Fig. 4:**
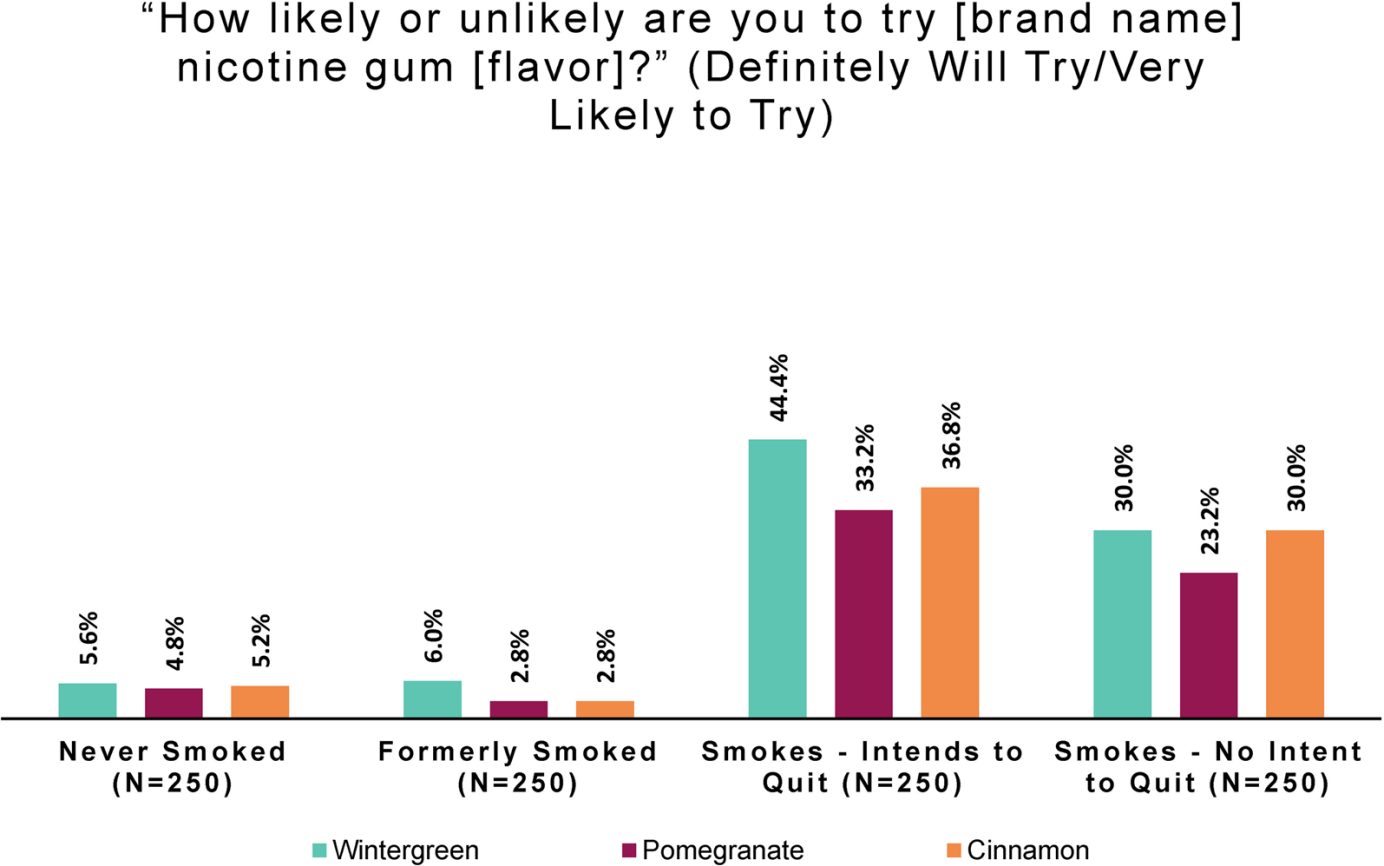
Positive intention to try: by smoking behavior and flavor (online panel)

As before, to facilitate comparison of subgroup responses, we derived a simple measure of intention to try. Positive intention to try combines the “very likely to try” and “definitely will try” responses. Results are shown in Fig. 4.

People who smoked show a high openness to trying the nicotine gum under study, a product that is presumably new to them. By contrast, only a tiny percentage of persons who never smoked or formerly smoked expressed a positive intention to try any study product flavor.

To assess the odds of intent to try the product by smoking behavior category, we constructed a variable that represents a positive intent to try one or more of the three flavors. Results are shown in Table 3.

**Table 3:** Positive intent to try any study product flavor, by smoking behavior

Smoking behavior	No	Yes	Total
Formerly smoked	232 (93%)	18 (7.2%)	250 (100%)
Never smoked	229 (92%)	21 (8.4%)	250 (100%)
Smokes-no intent to quit	142 (57%)	108 (43%)	250 (100%)
Smokes-intends to quit	113 (45%)	137 (55%)	250 (100%)
Total	716 (72%)	284 (28%)	1000 (100%)

Logistic regression was performed to express the odds of intention to try for each smoking behavior group, using persons who formerly smoked as our reference group. Table 4 shows the results. Those who formerly smoked have very little intention to try any study product flavor, as expressed by the small odds (0.08). By comparison, the odds of intent to try any product flavor among people who smoke and do or do not plan to quit are 15.6 and 9.8 times higher respectively. The odds for persons who never smoked are not significantly different from those who formerly smoked.

**Table 4:** Logistic regression analysis of positive intent to try any study product flavor

Smoking behavior	OR	95% CI	*p* value
Formerly smoked	–	–	
Never smoked	1.18	0.61, 2.30	0.6
Smokes-no intent to quit	9.80	5.84, 17.3	< 0.001
Smokes-intends to quit	15.6	9.32, 27.6	< 0.001

*OR* odds ratio, *CI* confidence interval

To aid in interpretation of these findings, we looked at comments left by study participants. The online panel survey included one open-ended question to elicit such comments: “Overall, what do you think about the [study product brand name] nicotine gum product?”.

Comments from persons who formerly smoked overall support a lack of interest and/or lack of perceived personal relevance of the product. Many brief comments were negative (e.g., “sucks,” “It seems ridiculous to me,”) or positive but impersonal (e.g., “concept is okay,” “it looks good hope it works”). Longer comments were along the same lines:

[Age 25–29]: “I think it looks like a very nice product from the packaging and I think people would use it, I would recommend it.”
[Age 25–29]: “I already quit smoking so I won’t be trying but could be helpful to others.”
[Age 30–34]: “Looks tasty, but I don’t need it.”

Similarly, comments from people who had never smoked were either negative or detached in tone.

[Age 21–24]: “It looks interesting and discreet, and like it could be helpful to people looking to stop smoking.”
[Age 21–24]: “not applicable to me but good idea.”
[Age 25–29]: “I do not like the thought of anything having nicotine in it. I do not like it.”
[Age 55–64]: “I’m sure it serves a purpose but it would be addictive. I suppose that better than sucking smoke into your lungs.”

A respondent who did not smoke noted, “honestly not bad, looking to quit chewing [tobacco] and might try it.”

Among people who smoked but intended to quit within the next 6 months, many appeared to perceive the study product as a means to quit or reduce smoking. Typical comments were positive but brief, along the lines of “sounds interesting,” “it’s worth a shot,” or “would like to try it.” Some were skeptical, e.g., “Like trading vices.”

Many of the detailed comments specifically mentioned the product in the context of quitting. For example:

[Age 21–24]: “I think that it is a good product for folks who are serious about quitting smoking. I do think that people could start to use this as their ‘vice’, though since they are still regularly taking nicotine into their bodies.”
[Age 25–29]: “I would try it as a start to quitting the real thing.”
[Age 25–29]: “I like the look and what the product has to offer. This product would motivate me to quit smoking.”
[Age 45–54]: “If it could reduce the amount cigarettes i smoke the benefit would maybe outweigh the risks.”

From people who smoked and were not planning to quit in the next 6 months, many comments were brief but positive, e.g., “it might help,” “seems like a decent substitute,” or “I would try it.” A few responded with skepticism, e.g., “Another pointless product. The only way to quit is cold turkey.”

Other comments were positive but didn’t find the product personally relevant, e.g., “It seems good for those interested in this type of product.”

However, many persons with no plans to quit smoking appeared to find the study product appealing or expressed interest in trying the product. For example:

[Age 25–29]: “I like the idea that this product will freshen breath, keep your mouth busy whenever you get irritated or feel as though you need to smoke, easy to carry, and also doesn't come with the cigarette smell.”
[Age 25–29]: “I would definitely try due to my job and not having a lot of time for smoke breaks.”
[Age 45–54]: “It's a new way to get nicotine into your body without all the smoke think it's kind of great.”
[Age 55–64]: “[Brand name] sounds like a good product to help me quit smoking. I like the flavor choices.”

#### Product appeal and intent to try among young adults: online panel

**Fig. 5:**
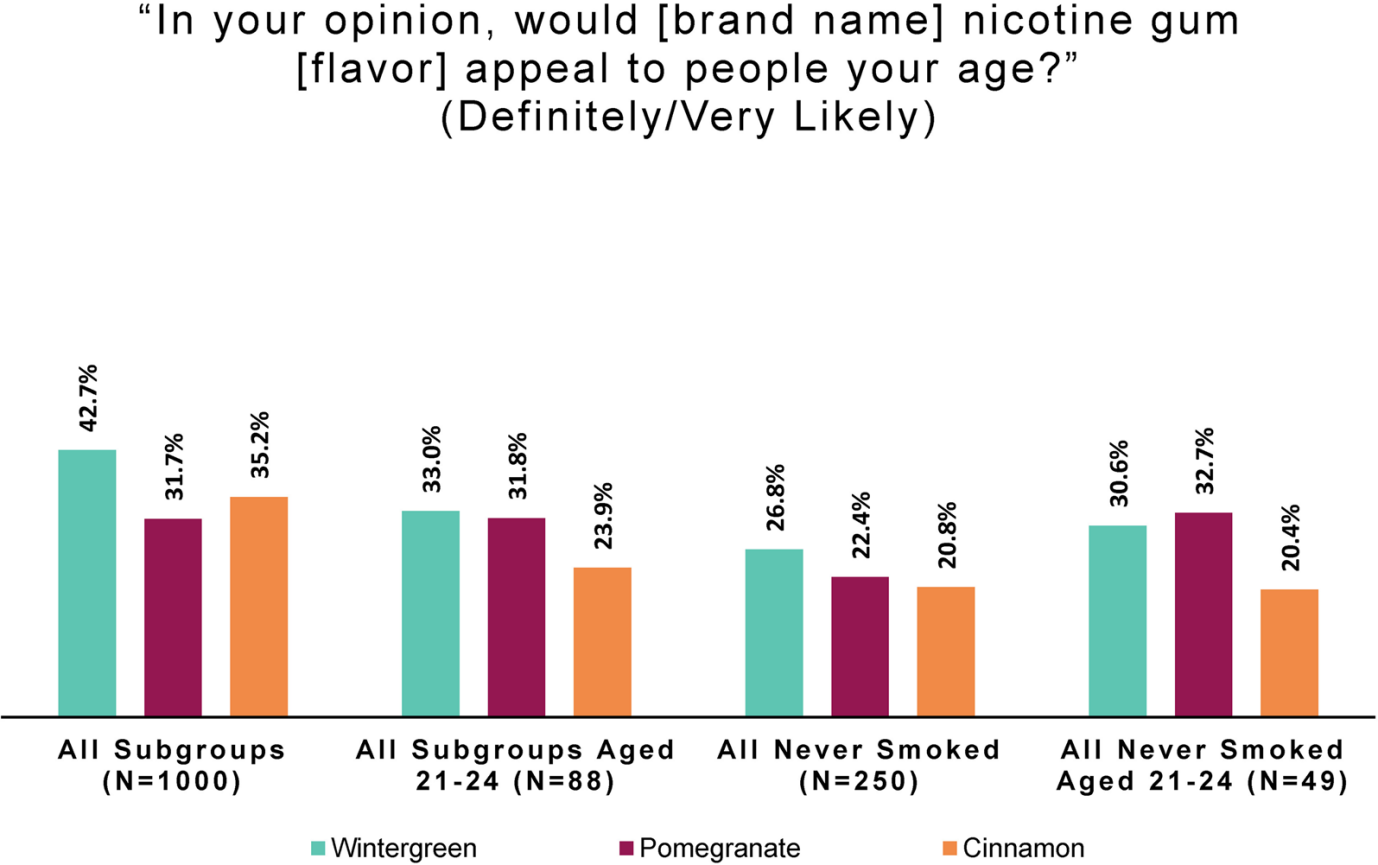
Positive age group appeal: by age group and flavor (online panel)

Product appeal and intentions to try among legal-age adults under 25 is viewed by U.S. regulators as an acceptable proxy for potential product appeal among underage youth since “inferences regarding individuals below the minimum age of sale may potentially be extrapolated from young adults” [8]. We looked at appeal of and intention to try the three product flavors among all subjects age 21–24 (*N* = 88) and those who had never smoked aged 21–24 (*N* = 49) to see if their responses differed substantially from the total sample, and from all persons naïve to smoking in the sample. As in the above section, positive age group appeal was derived by combining responses that the product would “definitely” or “very likely” appeal “to people your age” (see Fig. 5).

Small cell sizes require approaching these data with caution. However, the product did not have more positive age group appeal among the youngest adults than among the total sample; results vary by flavor for the youngest adults naïve to smoking.

**Fig. 6:**
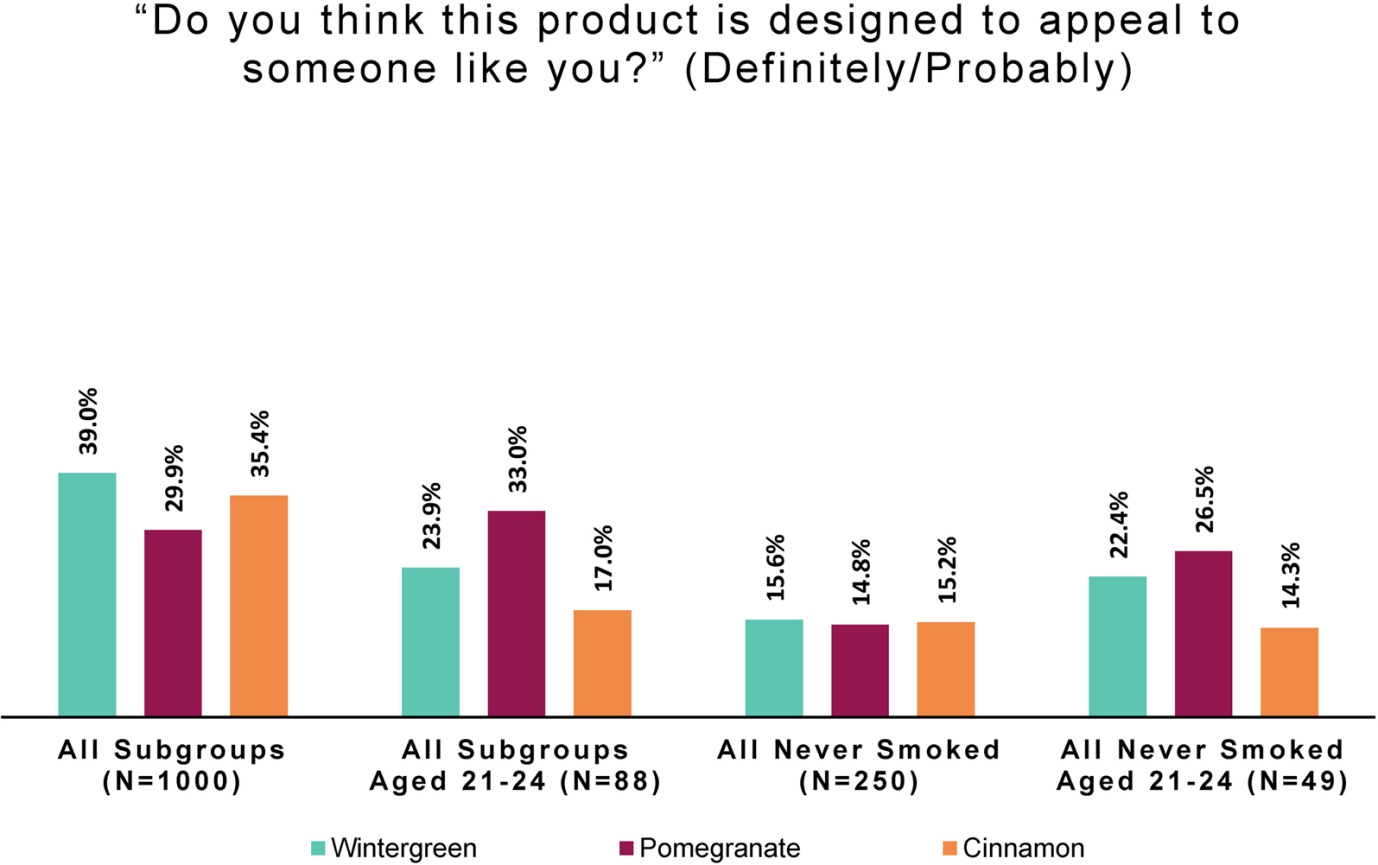
Positive personal appeal: by age group and flavor (online panel)

Figure 6 depicts personal appeal by flavor and age. As in the section above, positive personal appeal was derived by combining responses that the study product was “definitely” or “probably” designed “to appeal to someone like you.”

Similar to the total-group results, the most common response among young adults who had never smoked regarding whether any product flavor “was designed to appeal to someone like you” was “definitely not”: 32.7% for wintergreen, 42.9% for pomegranate, and 42.9% for cinnamon.

**Fig. 7:**
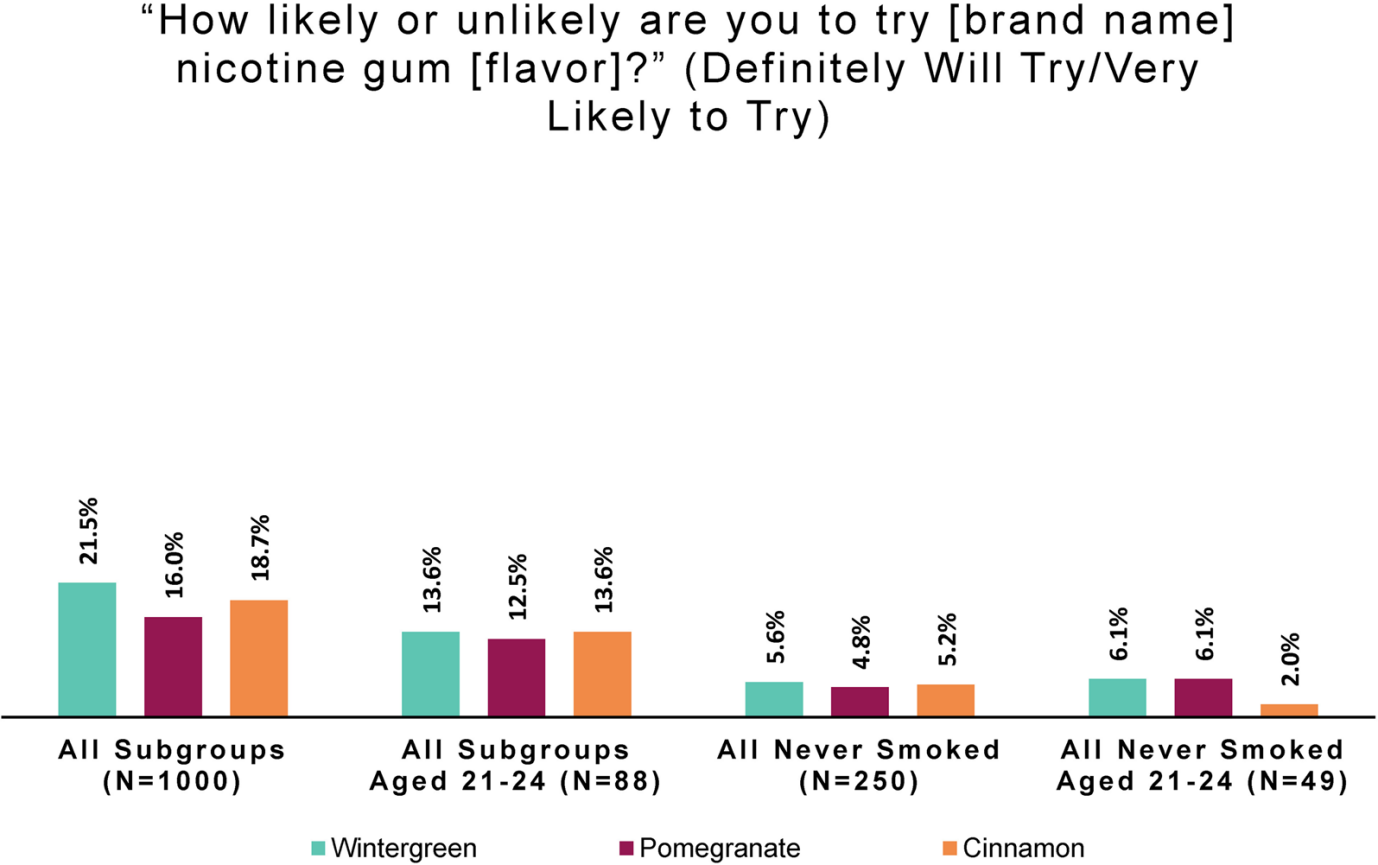
Positive intention to try: by age group and flavor (online panel)

Finally, we looked at positive intention to try any study product flavors among the youngest adults. As seen in Fig. 7, compared to the total sample, the youngest adults had *lower* positive intentions to try any product flavor. Looking only at those who had never smoked (who all have very low intentions to try), the youngest adults showed slightly higher interest in wintergreen and pomegranate and lower interest in cinnamon.

The 49 persons age 21–24 who had never smoked had a high “negative intention to try” any flavor. The proportion of this group indicating they “definitely will not try” or are “very unlikely to try” wintergreen was 83.7%; pomegranate was 85.7%, and cinnamon was 81.6%. These are higher negative intentions than were found for the total group of respondents who had never smoked (78.0%, 80.8% and 79.6% respectively).

#### Effect of exposure to product packaging on intention to quit smoking

As noted in the recruiting description, at the start of the survey 250 out of 500 persons who smoked indicated that they planned to quit (now or within the next 6 months). Asked the same question at the conclusion of the survey, 266 endorsed plans to quit. This suggests that exposure to information about the product and package images may not reduce intentions to quit smoking.

#### Risk perception

**Fig. 8:**
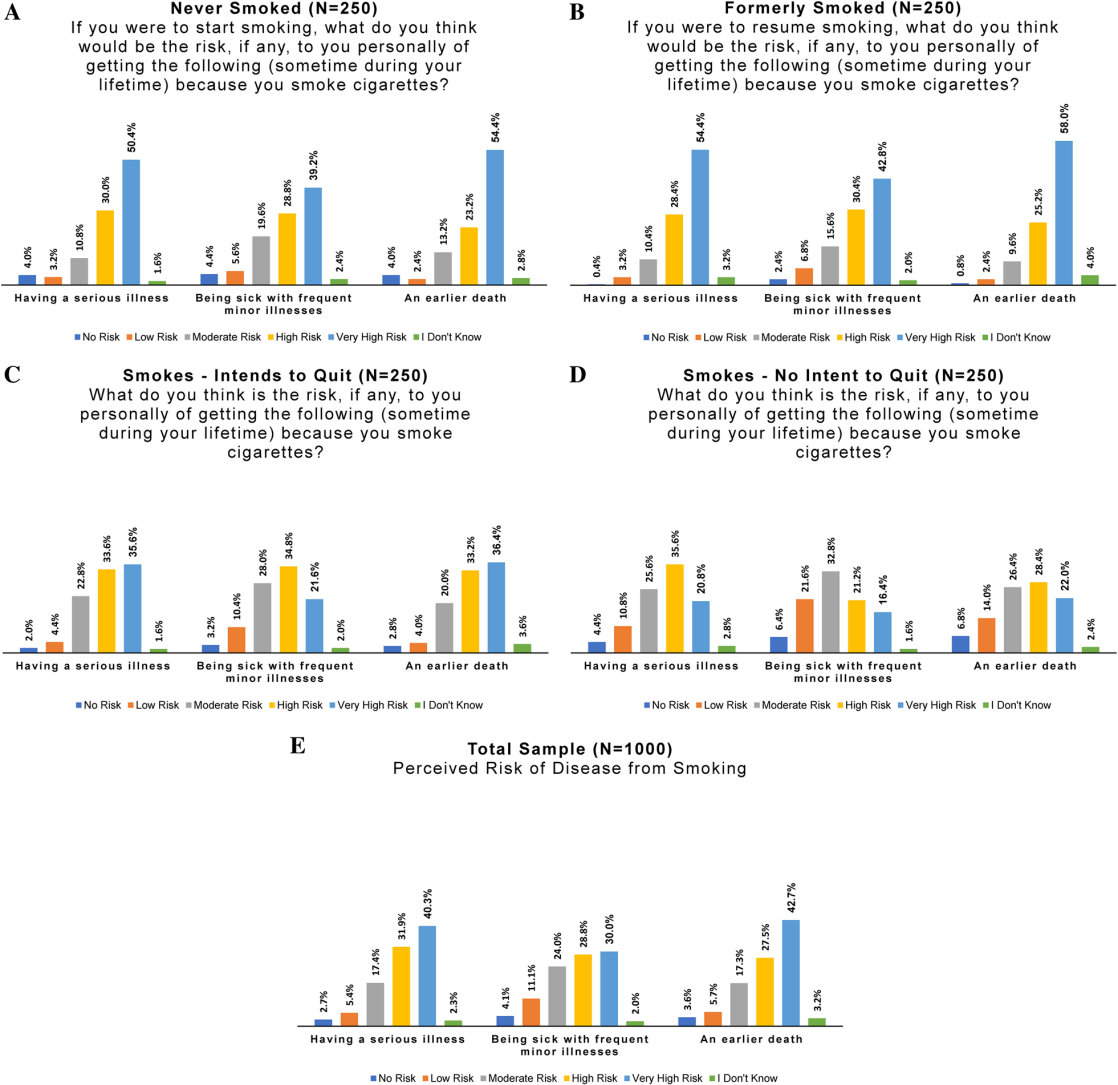
Perceived health risks of cigarettes: **A** Never smoked, **B** formerly smoked, **C** smokes—intends to quit, **D** smokes—no intent to quit, **E** all subgroups combined

To assess perceptions of *absolute* health risks of cigarettes and of the consumer nicotine gum by online panel members, we adapted several items from PMI’s Perceived Risk Instrument (PRI) [31]. PMI’s instrument included 18 items on perceived personal health risk plus two on harms to others. To reduce the response burden and increase validity of responses, we selected and combined several items with the goal of capturing a range of severity of perceived harm. The hypothetical harms included “having a serious illness,” “being sick with frequent minor illnesses,” and “an earlier death.” The version of questions presented to respondents varied based on their current smoking status. For example: “What do you think is the risk, if any, to you personally of getting the following (sometime during your lifetime) because you smoke cigarettes?” for people who smoked, and “If you were to start smoking, what do you think would be the risk, if any, to you personally of getting the following (sometime during your lifetime) because you smoke cigarettes?” for people who had never smoked (Fig. 8A–E).

All four online panel smoking behavior subgroups viewed cigarettes as carrying high risks of illness and death. However, risk perception was highest among those who formerly or never smoked, and lowest among those who smoked and did not intend to quit. For example, 80.4% of people who had never smoked perceived high or very high risk of having a serious illness due to smoking, compared to 82.8% of those who formerly smoked, 69.2% of persons intending to quit smoking, and 56.4% of persons not intending to quit smoking. Interestingly, a comparatively lower percentage of persons not intending to quit perceived high/very high risk of frequent minor illnesses from cigarettes (37.6%, compared to 56.4% for those intending to quit cigarettes, and 73.2% of those who formerly smoked). Only half (50.4%) of people who smoked and did not plan to quit perceived a high/very high risk of an earlier death from smoking, compared to 69.6% of those intending to quit, and 83.2% of persons who had previously stopped smoking.

**Fig. 9:**
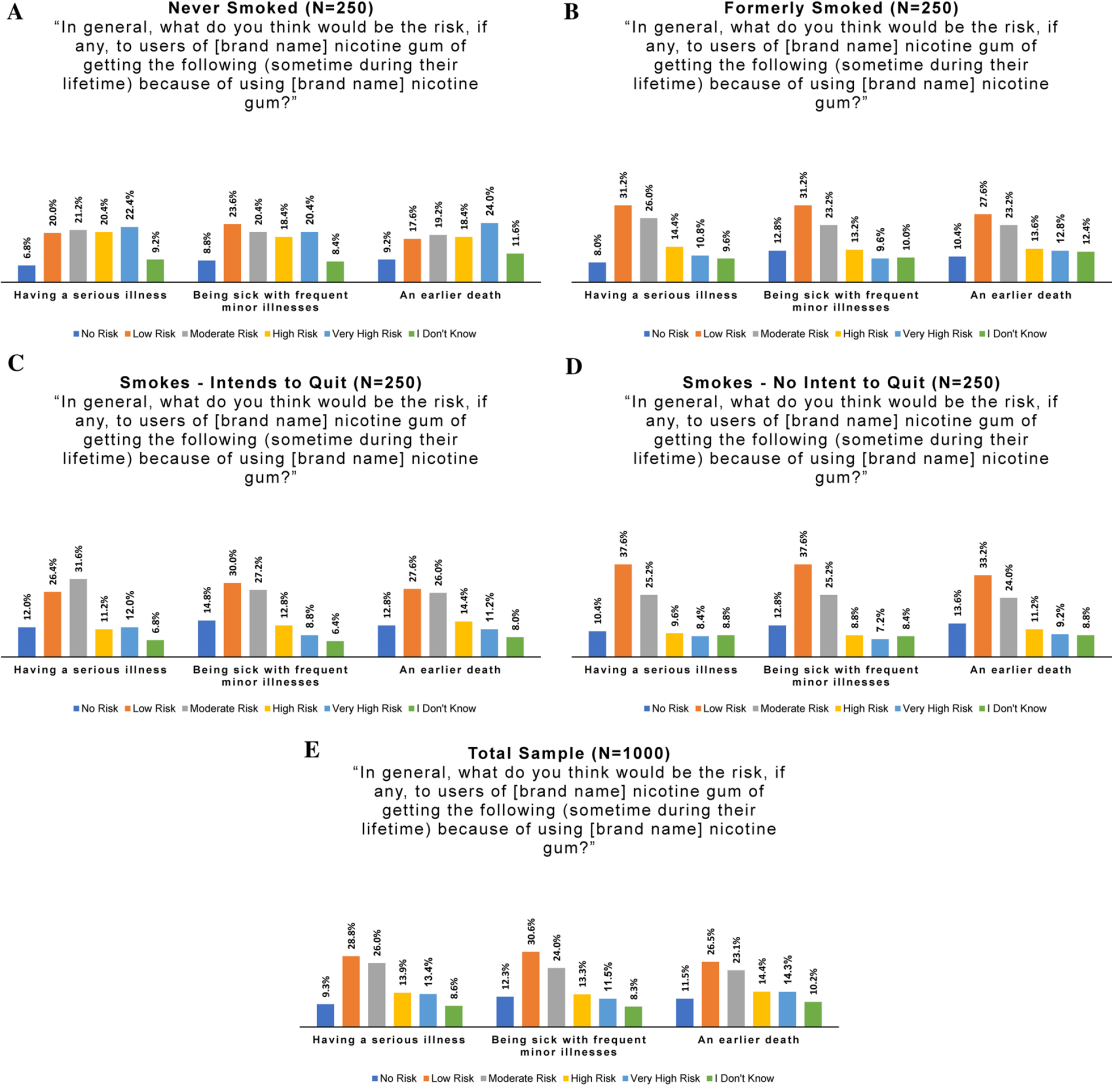
Perceived health risks of the study product: **A** never smoked, **B** formerly smoked, **C** smokes—intends to quit, **D** smokes—no intent to quit, **E** all subgroups combined

To assess and compare perceptions of potential health risks of the consumer nicotine gum product, a related set of questions was posed to all subgroups, as shown in Fig. 9A–E.

Results were also reviewed by smoking behavior category, to assess whether all subgroups (especially persons who now smoke) understood that the study product is not a substitute for cessation. Risk perceptions varied greatly among the four subgroups. The majority of all subgroups perceived the product as carrying at least some level of health risk, with fewer than 15% of respondents answering “no risk” for any question (fewer than 10% for those who never smoked). Half (48%) of people who smoked and did not intend to quit saw the study product as carrying no or low risk of causing a serious illness, but only a quarter (26.8%) of those who had never smoked saw no/low risk (versus 39.2% of those who formerly smoked and 38.4% of those intending to quit smoking).

A strikingly higher percentage of people who had never smoked perceived great risk of illness or death from the study product as compared to other subgroups. For example, 42.8% saw high to very high risk of having a serious illness due to use, compared to 25.2% of persons who formerly smoked, 23.2% of those intending to quit smoking, and 18.0% of those not intending to quit. Further, 38.8% perceived a high/very high risk of frequent minor illnesses (vs 22.8% of those who formerly smoked, 21.6% of those intending to quit smoking, and 16.0% of those not intending to quit). Finally, 42.4% of persons who had never smoked perceived a high/very high risk of an earlier death (compared to 26.4% of those who formerly smoked, 25.6% of those intending to quit smoking, and 20.4% of those not intending to quit).

Responses about the health risks of cigarettes and of the product also reflected a relative lack of knowledge about the study product and/or nicotine gum in general. Responses of “I don’t know” among subjects tended to range from 2 to 3% to questions about cigarette health risks, compared to 6–12% for the corresponding questions.

Relative risk perceptions were assessed for the consumer nicotine gum purchasers and for the online panel via a product ranking question, adapted from one described by Czoli et al. [26]. Instructions read, “Please rank the following tobacco products in terms of health risks. Use ‘1’ for the item that is the most harmful, ‘2’ for the 2nd most harmful item, and so on, up to ‘7’ for the item that is least harmful. Please rank all items. If you aren’t sure, make your best guess.” The “best guess” language was adapted from questions about risk perceptions of novel tobacco products used by Pepper et al. [32]. The question was programmed so that online survey respondents had to use numbers one to seven, and could use each only once. Items ranked, and their mean rankings, are presented in Table 5.

**Table 5:** Tobacco product risk rankings by study product purchasers and online panel: means and standard deviations (a higher value indicates a lower perceived risk)

	Study product purchasers		Online panel	
	Mean	Standard deviation	Mean	Standard deviation
Cigarettes	1.3	1.0	1.5	1.0
Chewing tobacco, snus, snuff or dip	2.3	0.9	2.2	1.0
E-cigarettes/vaping	3.0	1.1	3.3	1.6
Nicorette gum	4.5	0.8	4.3	1.1
Nicotine patch	4.9	1.0	4.7	1.1
Consumer nicotine gum (study product)	5.3	0.9	5.1	1.0
Using no tobacco products	6.7	1.1	6.9	0.4

Among online panel participants, all smoking behavior subgroups viewed “using no tobacco products” as least harmful (ranked #7 of #7 on risk). However, those who formerly smoked had the greatest consensus, with 97.2% ranking no tobacco as least harmful, compared to 85.2% of those intending to quit smoking and 87.2% of those not intending to quit.

All online panel subgroups ranked cigarettes as #1 most harmful, although this ranged from a high of 78.0% for persons who formerly smoked to a low of 58.8% for persons not intending to quit smoking. (Those intending to quit were in between, at 66.4%.).

Chewing tobacco was the next-most-frequent choice for #1 harmful tobacco product, with a quarter (24.4%) of persons not intending to quit smoking ranking chew #1. Notably, 15.6% of persons not intending to quit smoking ranked e-cigarettes as most dangerous, well above other subgroups.

On average, the study product was ranked #6 out of 7 in terms of perceived harm by online panel members, but there was a range of opinion. Interestingly, more persons not intending to quit smoking (46.0%) ranked the study product #6 (less harmful than other products but more harmful than quitting tobacco) than did any other subgroup—even more than did study product purchasers (45.2%). Online panel members who had never smoked showed the least consensus about the safety of the study product, with 36.4% ranking it #6, 33.6% ranking it #5, and 22.4% ranking it the #4 most harmful tobacco product.

### Tobacco product perceptions, intentions and actual use by consumer nicotine gum purchasers

#### Risk perception

When study product purchasers were asked to rank a list of tobacco products in terms of health risks, cigarettes were ranked most frequently as #1 most harmful (81.6%), with chewing tobacco a distant second (9.8%). Just 0.4% (two people) ranked the study product as most harmful. “Using no tobacco products” was ranked least harmful by 92.8%; 2.4% ranked the study product as least harmful.

#### Motivations for initiating

All consumer nicotine gum survey respondents were asked about reasons for choosing to try the study product, with multiple responses permitted*.* Seven response options were provided, along with "other.” “To help me quit e-cigarettes/vaping” was selected most frequently, by 41.0%. Nearly a quarter (23.4%) wanted help to quit smoking (including 43.4% of those subjects who reported smoking regularly before trying the study product). Also, 30.6% wanted a product to use where smoking is not possible or permitted (implying some movement toward dual use). Other motivations included curiosity (36.8%), “wanted a product to help me focus” (27.4%) and wanted an energy boost (18.2%). Fifty-two respondents wrote in an additional reason; 26 of these comments (5.2% of respondents) mentioned cutting down or quitting some form of oral tobacco use (e.g., “To help me quit dipping.”).

Curiosity is one factor often assessed in youth surveys as possibly representing future susceptibility to experiment with tobacco products [33]. Interestingly, a slightly smaller percentage of the youngest adult respondents (aged 21–24) to the consumer nicotine gum survey cited curiosity as one of their motivations for trying the study product than did the next-oldest cohorts. Specifically, 38.8% of subjects aged 21–24 marked “curiosity” when asked why they chose to try the product, versus 39.2% of subjects aged 25–30 and 42.9% of subjects aged 31–34.

#### Daily product consumption

The 294 persons who currently purchased the study product were asked, “On days that you use [study product brand name], about how many pieces do you chew?” Most common was 3–5 pieces per day (45.6%); others reported 0–2 pieces (39.8%), 6–8 pieces (9.9%), or 9–11 pieces (4.1%). Two people reported chewing 12 or more pieces per day. Daily use was heavier among the 62 persons who reported trying the consumer nicotine gum to quit smoking; 61.3% used 3–5 pieces per day, and 19.4% consumed more or less than that amount. (Although the manufacturer does not provide suggestions on daily use amounts, these figures are well within the use range suggested for, and similar to, that of NRT gum. Daily use was also below the advised maximum daily consumption of 24 pieces for 4 mg nicotine polacrilex gum used as NRT.)

#### Perception of product characteristics and role in satisfaction

To understand why purchasers might choose the study product (i.e., nicotine gum) over other nicotine products, they were presented with a list of six characteristics (plus “other”) and asked which of them they liked about using the product. Both current and former product purchasers most often chose “That it’s discreet to use (no smoke or smell)” (71.2%, 67.7%). For current purchasers, “The nicotine effect (‘buzz’)” was the second most frequent choice (65.9%); by comparison, 48.0% of former buyers chose “buzz.” Flavor and taste appeared important to the choice to use the study product; “How it tastes” was chosen by 64.9% of current and 59.6% of former purchasers, and “The flavors available” by 58.6% of current and 59.1% of former purchasers. The most commonly selected disliked feature (out of six) about the product’s taste or texture was how long the flavor lasts (21.8% current purchasers, 26.5% former buyers).

The importance of flavors was also supported by responses to the online panel survey of persons naïve to the study product. An open-ended question that asked, “Overall, what do you think about the [study product brand name] nicotine gum product?” drew 63 comments that included flavor or taste, e.g., [Person not planning to quit smoking, age 55–64] “might try it myself because of the flavors offered,” [Person intending to quit smoking, age 21–24] “I think the idea is good, but I would want to see reviews about how it tastes,” and [Person intending to quit smoking, age 65–74] “Sounds a whole lot better than Nicorette, which tastes awful!”.

To understand relative appeal of the study product versus NRT gum, study product purchasers who had also used or tried Nicorette® gum (*N* = 253) were asked similar questions about Nicorette®. The primary difference appeared to be perception of product taste. Only 27.3% indicated they liked Nicorette’s taste, and 19.8% liked the flavors available. Similarly, in an open-ended question about how the study product compares to Nicorette® gum, the most frequent topic among 185 comments was taste or flavor, with 63 of 70 such comments indicating a preference for the study product.

Asked what they did not like about how the study product made them feel, “The nicotine effect (‘buzz’) is too weak” was the most common response (31.1% of current study product buyers, 33.8% of former). Few found the nicotine effect too strong (7.0% current purchasers, 9.1% former). By contrast, only 16.6% of those who had tried Nicorette® chose a too-weak nicotine effect as something they had not liked about that product.

The most-selected undesirable physical effects from the study product were “Upsets my stomach” (17.9% current purchasers, 22.2% former), “Caused discomfort in my mouth, throat, or teeth” (15.9% current, 22.2% former), and “Caused coughing or hiccups” (13.9%, 11.6%). This compares to slightly higher percentages who chose upset stomach (26.1%), mouth discomfort (31.2%), and coughing/hiccups (17.0%) as effects they disliked from using Nicorette®.

To assess whether the “medicinal” image of NRT gum might affect the choice to purchase a consumer nicotine gum product, one response option in the list of potential negatives for both the study product and Nicorette® was “It doesn’t [didn’t] feel like a product for someone like me.” Interestingly, there was little difference in responses for study product purchasers (2.0% current, 9.1% former) and Nicorette® purchasers (8.7%). Some consumer comments comparing the study product and Nicorette® suggested that product image was relevant for them, e.g., [Age 25–30] “The appearance of the product is bold and modern compared to the bland Nicorette gum,” [Age 35–44] “[brand name] is in a more discreet packaging and feels like chewing gum instead of nicotine gum,” [Age 35–44] “[brand name] is not as medical tasting,” and [Age 31–34] “It seems ‘cooler’—Nicorette feels embarrassing, like something older people use.”

#### Typical product consumption situations

Current (*N* = 294) and former (*N* = 186) study product purchasers were asked in what situations they typically use(d) the product. (Twenty subjects who only tried the product once or twice were excluded.) The most common responses for both groups were “I use(d) it throughout the day to prevent nicotine cravings” (42.9% and 50.5% respectively), “When I feel (felt) a nicotine craving coming on” (45.6% and 46.8%), and “When I want(ed) to focus” (44.2%, 29.0%). People who initiated consumption to quit smoking were especially likely to choose preventing nicotine cravings (75.8% of 62 current purchasers, 64.0% of 50 former) and coping with oncoming cravings (56.5%, 64.0%) as situations when they use(d) the product.

Respondents also turned to the study product when they were feeling stressed or down (34.4%, 23.7%), feeling bored or wanting a break (23.5%, 18.3%), feeling tired (24.1%, 17.2%), socializing with friends (17.3%, 8.1%), or feeling hungry (9.2%, 8.1%). Other reasons written in focused on discreet consumption, such as “Situations where it’s inappropriate to vape, i.e. work and around my child,” “When I didn’t want to smell of smoke,” “When I don’t want to offend others with my Vape,” and “When I ran out of smokeless tobacco, or when I needed nicotine discreetly.”

#### Products current consumers would switch to if the study product was not available

To better understand what nicotine products this consumer nicotine gum was perceived to substitute for, current purchasers were asked “If [study product brand name] wasn't available, what product(s) would you most likely use instead?” More than one response was permitted. Among 212 current purchasers who identified as male, the most common alternative choices were e-cigarettes/vapes (47.6%), Nicorette® gum (32.1%), chewing tobacco, snus, snuff or dip (20.8%), nicotine pouches (19.3%), or cigarettes (15.6%). Nicotine lozenges were listed by 11.8%; the patch, spray, and cigar/cigarillo/pipe options were chosen by 7–8% each. The 74 current purchasers who identified as female most often named Nicorette® gum (32.4%), e-cigarettes/vapes (29.7%), cigarettes (23.0%), or nicotine lozenges (16.2%). The nicotine patch was chosen by 8.1%, pouches by 5.4%, and other options by less than five percent of women. In contrast to one-fifth of males, no female current purchasers indicated that they would switch to traditional smokeless tobacco.

Of the 147 current product buyers who had smoked regularly before trying the product, one third (32.6%) would turn to cigarettes if the study product were unavailable. In addition, 44.2% would vape, and 34.0% would turn to NRT gum.

#### Intentions to employ the study product to cut down or quit smoking

**Fig. 10:**
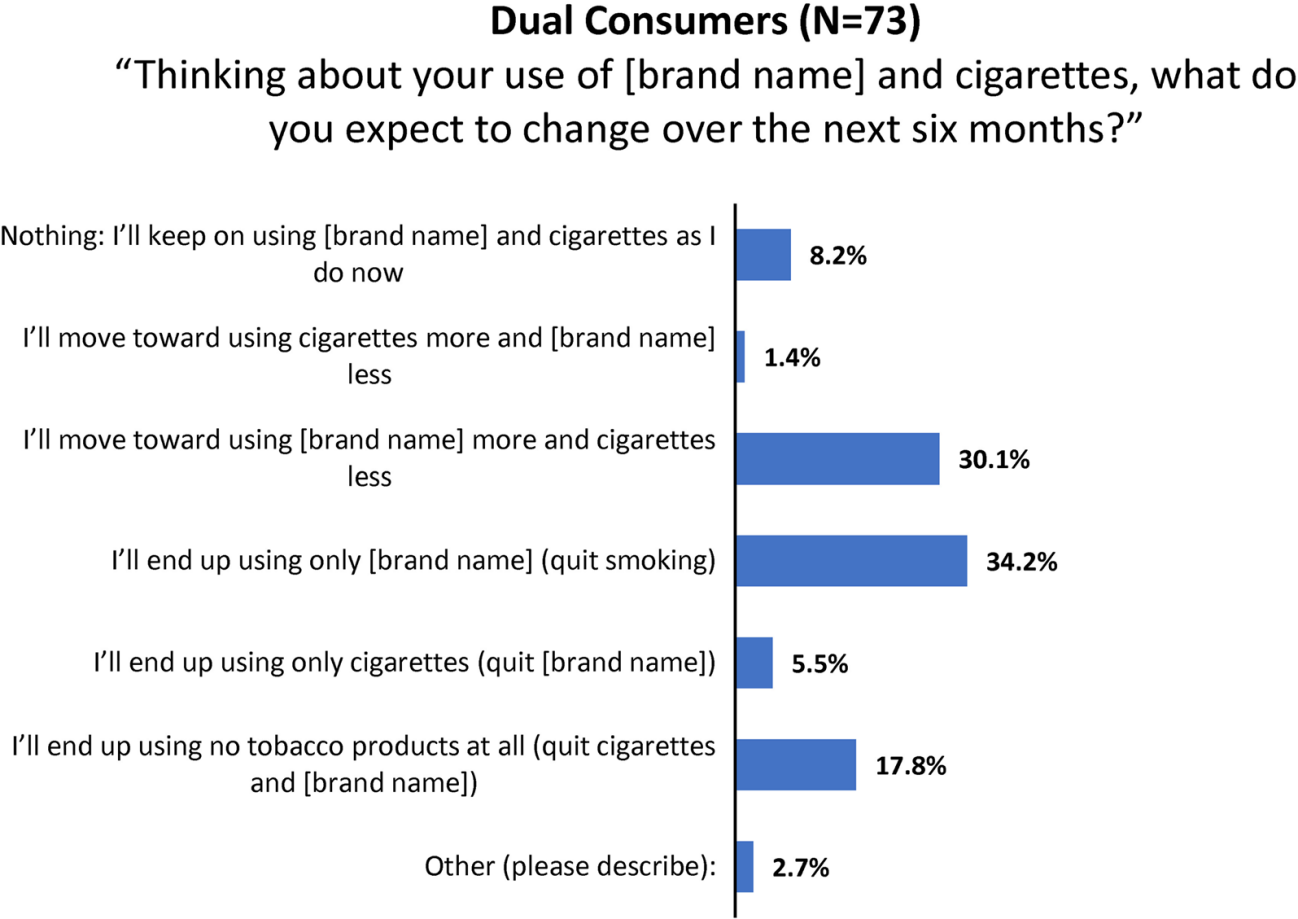
Consumer nicotine gum survey: intentions for dual use and quitting

Of 294 current product purchasers, 73 indicated that they currently smoked every day or some days. These respondents were asked about what they expected to change over the next 6 months regarding cigarettes and the study product. As shown in Fig. 10, over half (52.0%) of purchasers who smoke expect that in 6 months they will have quit smoking and be employing the consumer nicotine gum only, or no tobacco products at all.

#### Experience with the study product to cut down or quit smoking or vaping

**Fig. 11:**
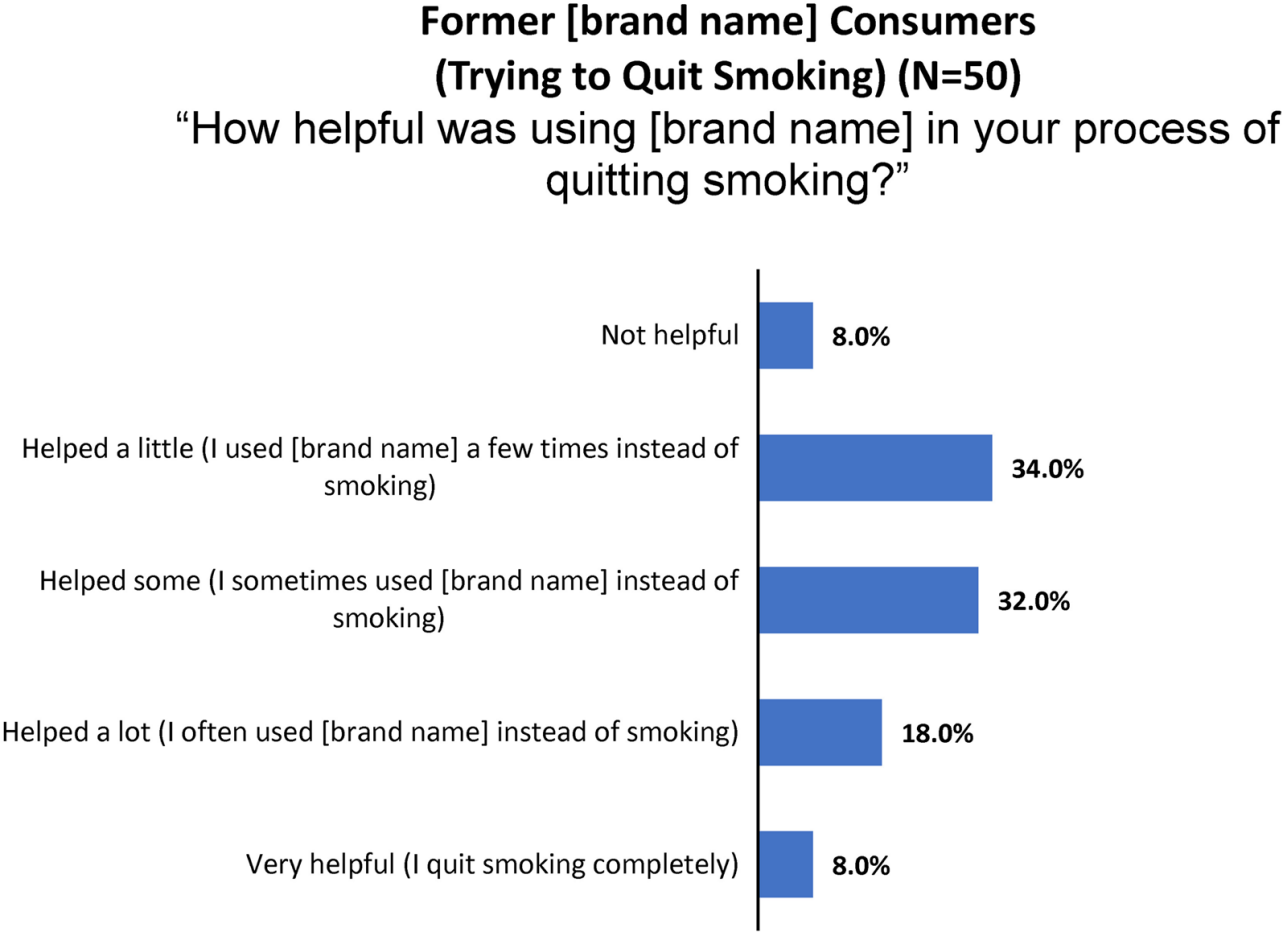
Consumer nicotine gum survey: reports of smoking cessation experiences

Former purchasers who indicated they chose to try the study product “to help me quit smoking cigarettes” were asked how helpful the nicotine gum had been in carrying out that intention (Fig. 11).

**Fig. 12:**
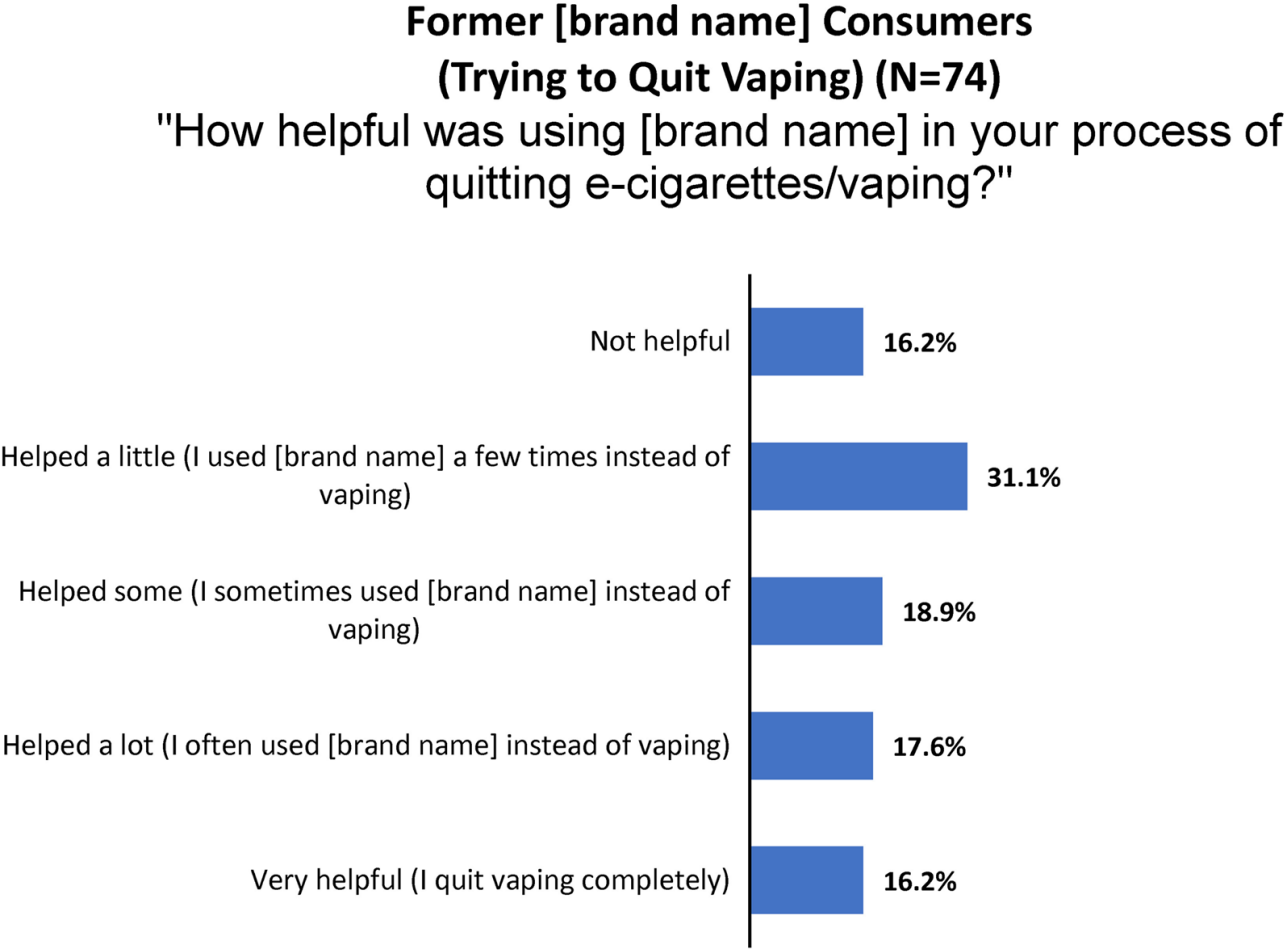
Consumer nicotine gum survey: reports of vaping cessation experiences

A similar question was asked of former purchasers who chose to try the product to quit vaping (Fig. 12).

The 111 subjects who initiated consumption to quit smoking (*N* = 50) and/or vaping (*N* = 74) were asked, “Please tell us more about your experience using [study product brand name] to cut down or quit.” All but two left interpretable comments.

People who smoked and found the product “helped some” (*N* = 16), “helped a lot” (*N* = 9) or was “very helpful” (*N* = 4) most often cited help with cravings and gradually cutting down smoking.

[Age 31–34]: “It helped me go from one pack a day to one pack every few days.”
[Age 21–24]: “It helped ease the feeling of wanting to smoke after having it in a while. it would hold me over until I was able to smoke, then I would take [brand name] out, smoke, and put a piece in the next time I felt like I wanted to smoke.”
[Age 65–74]: “I replaced a cigarette with [brand name] sevetal [sic] times per day at first then eliminated 2 cigarettes a each day until i quit.”

For those persons who smoked and indicated the product “helped a little” (*N* = 17) or was “not helpful” (*N* = 4), factors most often mentioned were lack of enjoyment (including finding the product bitter or too strong), adverse experience, cost, or insufficient help with cravings.

[Age 25–30]: “It killed the cravings but didnt enjoy the product.”
[Age 25–30]: “The gum was good, just gave me a weird feeling in my mouth that was not savory due to the nicotine. I may give it another try in the future.”
[Age 35–44]: “i liked the flavored (except mint). It did help. I just need to prioritize quitting.”

Among the 25 comments from people who vaped and found the product “helped a lot” or was “very helpful,” the most common theme was its successful use to combat cravings and gradually cut down on vaping.

[Age 31–34]: “Every time I had the itch, either I was able to hold off for an hour and see if it resumed—or, when stressed, took a piece of [brand name]. It wasn't a rush as much as it was a gradual cessation of the urge to vape.”
[Age 21–24]: “I started with vaping half of the time, and then using [brand name] the other half of the time. I kept cutting back on vaping and increasing my [brand name] usage till I completely stopped vaping.”
[Age 21–24]: “By using [brand name], I found that vaping was more of a habit and less of an addiction for me.”

Among people who vaped and indicated that the product “helped a little” (*N* = 23) or was “not helpful” (*N* = 12), the most common reason given was a perceived too-low nicotine dose or insufficient help with cravings. Fewer mentioned stomach or mouth discomfort, or perceived high cost.

[Age 21–24]: “[Brand name] didn’t give me the same buzz as a vape.”
[Age 25–30]: “I didn’t feel like it was strong enough to take the craving away.”.
[Age 21–24]: “I feel as if I am more addicted to the aesthetics of vaping than the nicotine rush itself. I tried [brand name] and still had the desire to blow smoke from a vape.”
[Age 25–30]: “I think [brand name] is a good product, I personally couldnt continue use because it would make my gums and teeth hurt. Even with a low nicotine dosage.”

#### Comments on experiences with the study product

The final question in the consumer nicotine gum survey asked, “Is there anything else you’d like to tell us about your experience using [study product brand name]? How has [brand name] affected your life?” A total of 211 subjects left substantive comments. Of these, the most frequent type (*N* = 67) concerned using the product to reduce or quit consumption of other tobacco products, including cigarettes (*N* = 27), e-cigarettes (*N* = 26), and/or chewing tobacco (*N* = 7). Below are several representative comments:

[Age 21–24]: “[Brand name] is one of the first products I tried when I wanted to quit smoking and it has helped me want a better life for myself so i'm grateful for [brand name].”
[Age 25–30]: “I used [brand name] gum to quit vaping. And haven't used a vape in over a month so I am very happy with the product.”
[Age 25–30]: “[Brand name] helped me to finally quit nicotine after 15 years of addiction. Thank you for making an amazing and effective product!”.

## Discussion

### Risk perceptions and misconceptions about nicotine

Responses to the risk perception questions in the consumer nicotine gum survey and the online panel survey were similar to results reported in other studies: combustible cigarettes were viewed as most risky, vaping and NRTs less so [26, 34, 35]. It is noteworthy and not clear why NRT gum and patches were perceived as riskier than the study product by online panel members. One possibility to investigate is whether a consumer nicotine gum product is less likely to trigger an exaggerated risk perception than a product packaged as a medicine.

Package design^1^ that appears similar to traditional chewing gum could affect risk perception [36]. This could be a positive if familiarity encourages people who smoke to trial the product, but could be a negative if it encourages nicotine-naïve persons to initiate. The product’s distribution primarily via online subscription sales, and prominent display of a nicotine warning on the front of packaging, may mitigate the latter concern.

A review of factors affecting patient adherence to NRT [37] found that perceptions of NRT safety and efficacy may play a role in its effective use. For example, the review found that product safety concerns about NRT gum were linked to fewer daily pieces used and shorter gum treatment duration. Another review of research, on consumer perceptions of differences in risk across nicotine products [26], found that (compared to general population samples) “adult smokers greatly overestimated the relative risks of NRT.” In other words, people who smoke may avoid or limit their consumption of NRT gum because of their perceptions of the product’s safety.

Misperceptions about the risks of nicotine (separate from the products of tobacco combustion) are widespread, even among physicians [38]. Efforts to educate health professionals about nicotine and about the existence of the growing variety of novel smokeless alternatives could encourage trial of lower-risk nicotine products by people who smoke and are not ready to quit, feel discouraged by previous failed quit attempts, or distrust NRT. Historically, most people who attempt to quit smoking have done so without using pharmaceutical or behavioral support [39]. A 2019 Cochrane review [40] summarizing the modest success of nicotine replacement therapies (including NRT gum) for smoking cessation noted that the data reviewed applied only to people who were motivated to quit smoking, and that there is a need for more data on different ways NRT can be used to maximize success.

### Encouraging quit attempts, including among people who expect to continue smoking

Stubbornly high rates of smoking in the U.S., most noted among low-income vulnerable populations [41], have been blamed in part on the need for more programs or products that increase quit attempts, and low acceptance and general use of evidence-based (such as NRT) treatments among those people who do attempt to quit smoking [42]. Many consumer nicotine gum survey participants described a pattern of gradually reducing their cigarette and/or e-cigarette consumption. A large 2009 placebo-controlled double-blind randomized trial of 2-mg and 4-mg NRT gum (Nicorette® “original” flavor) [43] looked at quitting smoking via gradual reduction: replacing cigarettes over time with NRT gum. The authors found that even with no instruction in or behavioral support for quitting provided, persons preferring to quit smoking gradually “could substantially increase their success” (p. 103) by using nicotine gum. Moreover, they noted contradictory research findings suggesting that persons who plan to quit smoking via gradual reduction would be expected to have lower quit rates than those who stop smoking abruptly. Those who cut down gradually were less motivated to quit, and were less than half as likely as those who quit “cold turkey” to even make a quit attempt.

Finding ways to increase nicotine gum appeal and reduce factors linked to gum discontinuation could meaningfully affect harm reduction. Making sufficient use of NRT gum appears to increase success. In the 2009 study, subjects who chewed more than the median amount of gum in the first weeks were more than twice as likely to reach 28-day continuous smoking abstinence.

### The role of flavors in appeal

Consumer nicotine gum such as the brand studied has the potential to attract or sustain effective use by some proportion of those who found NRT gum unsatisfying. Research suggests that people who smoke and initiate consumption of NRT gum typically fail to continue long enough to benefit from it as a cessation aid [37, 44]. Flavors/taste was the most-mentioned differentiating factor between the study product and NRT gums by respondents who had tried both. A highly palatable (i.e., flavorful) consumer nicotine gum product might help prevent a return to smoking (relapse). Research on NRT suggests that nicotine gum can be effective in preventing lapses if persons who formerly smoked, or occasionally do, could be encouraged to consume it more often when faced with cravings to smoke [45].

This emphasis on adult appeal of flavors stands in contrast to popular opinion currently viewing flavors through the lens of attracting youth. The limited research available [46, 47] suggests little interest in NRT gum among youth and young adults. However, care must be taken to educate the public about the existence and intended uses of consumer nicotine gum and other novel smokeless products, as well as to distinguish them from products approved as nicotine replacement therapies [48], to prevent the growth of misconceptions which might undermine the opportunity for people who smoke to benefit from the harm reduction potential of both categories of products (NRT and consumer).

Areas for further research include whether a consumer nicotine gum such as the product studied can encourage more sustained use compared to NRT gum, and (building on comments to this effect from purchasers) whether consumer nicotine gum may be helpful in preventing relapse among persons who formerly smoked. There is also a need for additional research on the entire range of novel smokeless products, few of which have been assessed in peer-reviewed publications or included in ongoing surveys of tobacco product consumption.

## Conclusions

The majority of respondents in the perception, intention and actual use study of study product purchasers were existing consumers of tobacco products. Half of the respondents smoked regularly, and nearly two-thirds vaped regularly upon initiating trial of the study product. Quitting or cutting down consumption of other tobacco products, especially vaping and smoking, appeared to be the most common motivation for choosing to try the study product.

Many purchasers reported that the study product was helpful in preventing or managing nicotine cravings, or meeting needs that they formerly turned to other forms of tobacco to meet, such as managing stress or maintaining focus. Some persons who smoked reported successfully reducing their cigarette consumption and transitioning to the study product. Some transitioned completely from cigarettes to exclusive use of the study product or to quitting all tobacco products. This was also true of respondents who vaped.

Many purchasers reported intentions to switch to higher-risk tobacco products if the study product were not available. For example, one-third of current gum purchasers who had smoked regularly before trying the study product indicated they would return to smoking if the product were not available. Several recent studies have shown that limiting choices of alternative nicotine products for adult tobacco consumers could potentially result in switching to higher-risk products [49–51].

In results for online panel members naïve to the study product, the nicotine gum was not viewed as appealing by persons who did not smoke. This was also true for the youngest legal-age adult cohort, suggesting that the consumer nicotine gum is unlikely to attract youth. Similar findings with subjects who formerly smoked suggest that the product has low potential to promote relapse to nicotine use. Conversely, the product showed high appeal and intention to try among respondents who smoked and were planning to quit, as well as those not intending to quit. Exposure to the product description and packaging did not reduce quit intentions among those persons planning to quit smoking.

These results add to the limited body of research on novel smokeless nicotine products.

## Data Availability

The datasets generated during and analyzed during the current study are available from the corresponding author on reasonable request.

## Abbreviations

U.S. FDA: U.S. Food and Drug Administration
HPHCs: Harmful and Potentially Harmful Constituents
HINTS: Health Information National Trends Survey
NNN: *N*-Nitrosonornicotine
NNK: Nicotine-derived nitrosamine ketone
NRT: Nicotine replacement therapy
PMI: Philip Morris International

## Footnotes

1 See lucy.co for current images of product packaging.
